# The Unmet Need for Artificial Saliva

**DOI:** 10.1021/acscentsci.3c00803

**Published:** 2023-07-13

**Authors:** Alla Katsnelson

Most of us are awash in saliva: we produce and swallow about a liter of it each day, totaling more than 20 000 L in a lifetime. This substance plays many roles in our everyday lives. It enables us to swallow, helps us taste our food, and provides a first line of defense against pathogens. And by keeping our vocal cords, mouths, and lips lubricated, saliva enables fundamentally human capacities such as speaking and smiling.

Yet we often take the stuff for granted, smearing its reputation by calling it drool, dribble, spit, or slobber. “Its value only becomes apparent when you don’t have it anymore,” says Stefan Ruhl, an oral biologist at the University at Buffalo.

More than 1 in 10 adults experiences xerostomia—the sensation of dry mouth. It is most commonly caused by medication side effects and is therefore especially prevalent in people who take a panoply of drugs, usually older adults. It can also accompany diabetes and other autoimmune diseases as well as radiation treatment for head and neck cancers, which often damages salivary glands. Because spit plays many roles in our mouths, the consequences of xerostomia can include declines in dental health and even difficulty eating.

Although several products that call themselves artificial saliva have been available over the counter at pharmacies for decades, they provide limited relief. Recreating spit’s paradoxical texture—slippery but not slimy, runny but not watery—has proved difficult, and those dealing with xerostomia have long sought better options.

**Figure:**
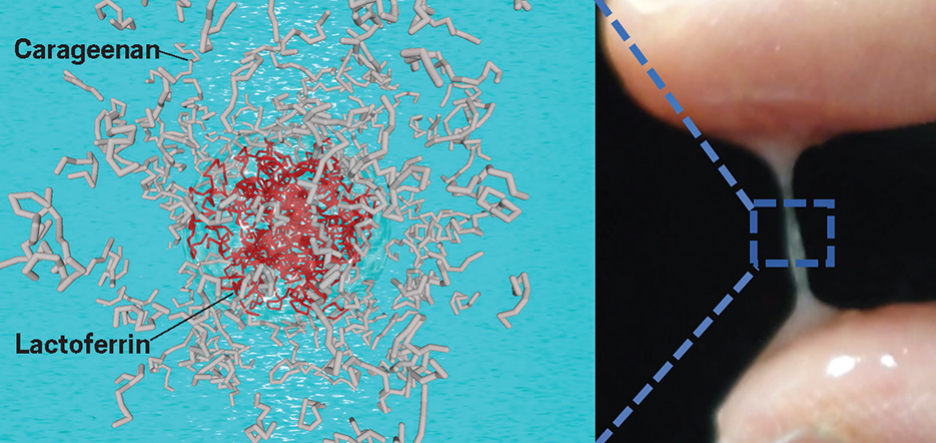
Anwesha Sarkar's lab combines lactoferrin microgels (red) with carrageenan hydrogels (gray) to produce a material with structural properties that recreates key properties of saliva. Credit: *ACS Macro Lett.*

Indeed, there is a significant and growing market for artificial saliva. A recent report by Data Bridge Market Research predicts it will almost triple, to $3.1 billion, by 2030. But on a commercial level, “there doesn’t seem to be much innovation in the field,” says Guy Carpenter, an oral biologist at King’s College London. The disconnect points to the chemical and material complexity of this humble body fluid.

Innovation may be on the way. Chemists are developing a broad suite of tools for characterizing saliva and other types of mucus in the human body and for recreating some of their key properties. While some research might facilitate efforts to make synthetic saliva, other research is taking a potentially cheaper and easier approach: supercharging the saliva that some people with xerostomia still produce. “It’s not a deadly disease, but it’s an annoying, quality-of-life-diminishing situation,” Ruhl says. “To recreate an artificial saliva is really a challenge, but it’s a challenge that’s really worth tackling.”

## Spit is spectacular

As a material, saliva is a substance awash in contradictions. For one thing, it consists of 99% water, but, despite water's association with creating slippery surfaces, it is the remaining 1% that performs saliva’s key function of lubricity.

Additionally, to work properly, saliva must adhere to the surface of the mouth, yet it must also form a nonadhesive surface to repel bits of food, bacteria, and whatever else finds its way past our lips. “It’s got to be like Teflon”—coat and stick to a surface but not stick to anything else, Carpenter says.

Saliva’s viscoelasticity is not straightforward either: it can vary dramatically over the course of a day, being more watery while someone chews and tackier when someone is at rest, says Anne Marie Lynge Pedersen, who researches saliva and the salivary gland at the University of Copenhagen and treats xerostomia as a clinician. “Even as we sit here and talk, our saliva is changing volume and changing in composition, depending on different stimuli,” she says. Different glands produce its more watery versus more lubricating components. “It’s a wonderful system when it works, but when it’s imbalanced, it’s very complicated to deal with,” she says.

Perhaps the most defining elements of saliva are proteins called mucins, which make up the bulk of the 1% of saliva that is not water. A handful of other players, including proline-rich proteins, agglutinins, and lipids, are more sparsely studied but also crucial to saliva’s properties.

Mucins exist in all kinds of mucus, and like many of our other sugar-laden glycoproteins, they are notoriously complex. Their shape, often likened to a bottle brush, consists of a long peptide backbone bejeweled with a slew of oligosaccharide side chains, each with defined and often branching structures. Those structures determine a given mucin’s consistency and other properties. The sugar chains also give the molecule a negative charge, which helps strings of mucins glide along one another and gives saliva its slippery feel and appearance. Most of the protein’s weight is often not amino acids but this dense forest of sugars sprouting from its backbone.

While this landscape of sugars is intricate, it is not random. The pattern and composition of sugars on each chain are largely responsible for why studying—and recreating—mucins has been so challenging. As a result, most artificial saliva products are viscous solutions with thickeners, such as carboxymethyl cellulose or poly(ethylene glycol), that merely mimic the tackiness that mucins confer on saliva. But these substitutes’ consistency feels abnormal in the mouth, so people generally do not like using them, Carpenter says.

“Saliva is not actually that viscous, and it’s not only viscosity that people are looking for—it’s hydration and lubricity,” says Anwesha Sarkar, a food and materials scientist at the University of Leeds. Mucins give saliva a peculiar texture: slippery but with a viscosity closer to that of water. “That is very unique,” she says.

What’s more, those artificial saliva products dissipate quickly in the mouth, so any relief they provide is fleeting. Concoctions that contain real mucins, usually extracted from pig salivary glands, work best, but those have downsides too: people tend to dislike the taste, and because of their source, they are off limits to some religious and cultural groups.

## Mimicking mucins

If chemists want to make artificial saliva from lab-made mucins, they need to invent better tools for studying the glycoproteins—tools that began to emerge only about 15 years ago, says Stacy Malaker, a chemist at Yale University who develops new methods for mucin analysis.

Even as recently in 2016, it was extremely challenging to analyze mucins with mass spectrometry because trypsin, the enzyme normally used to digest proteins for mass spec, could not chew through the thicket of sugars encasing the proteins. As a postdoctoral researcher in Carolyn Bertozzi’s Stanford University lab, Malaker characterized a family of bacterial enzymes called mucinases that could digest those sugar chains. This work opened the door for characterizing mucins with mass spec. At Yale, Malaker’s lab is using the approach to probe how different sugar patterns affect mucins’ functions and to explore how those patterns change because of disease. “If you’re going to re-create these things, it'd help to know what they look like in a biological system,” she says.

Some researchers are already using that emerging knowledge to create new mucins in the lab. “Part of the challenge in making artificial [saliva] is we didn’t have the chemistry to build things from the ground up,” says Jessica Kramer, a biomedical engineer at the University of Utah.

Kramer’s bottom-up approach first adorns loose amino acids with custom sugar chains and then strings them together to build a custom glycoprotein with high levels of complexity and control. Because this polypeptide synthesis technique is already used industrially, it could be scaled, she says, “and because we attach the sugars to the amino acids ourselves, we know exactly what’s there.”

Recently, the researchers synthesized mucin-like molecules from d-amino acids, mirror images of the naturally occurring protein building blocks. These nonnatural amino acids come together to make proteins that behave similarly to natural mucins and are hardier in a culture dish, likely because they are not recognized by microbial enzymes. “They can actually stick around a really long time, whereas regular mucins get devoured by bacteria,” Kramer says.

**Figure:**
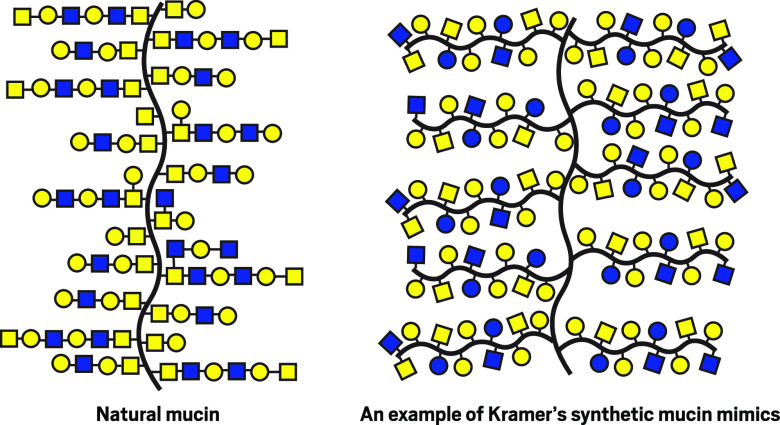
Native mucins (left) such as those in saliva get their lubricating properties from chains of various sugars (colored polygons) dangling off their peptide backbone. Jessica Kramer's group aims to mimic mucins with their synthesized glycopeptides that have individual sugar molecules attached to their branches. Credit: Jessica Kramer

Her lab has not focused on commercialization yet, but in principle the technique could be used to create a component of artificial saliva, she says. Synthetic substances mimicking saliva and other forms of mucus would also be valuable for modeling drug absorption through mucus layers, she adds.

But saliva has so many properties that recreating them all with a synthetic material may not be possible. Rather than build mucins from scratch, Matthew Paszek bestows cells with a desired mucin gene, creating factories that pump out the proteins. “Cells are just exquisite reactors for making complex polymers,” says Paszek, a chemical engineer at Cornell University. His work focuses not on saliva but on a mucin called lubricin, which acts as a joint lubricant.

He and Heidi Reesink, a veterinarian at Cornell, are testing whether lubricin relieves osteoarthritis in dogs and horses—which develop the condition much like people do—with the long-term plan of moving the material toward clinical translation for humans. The same production process could theoretically be used to create salivary mucins, he says. “Once you figure out how to recombinantly produce and do the engineering for one, that understanding should translate to another.”

Churning these molecules out at scale will require some optimization. Although plenty of clinical-grade facilities can produce recombinant antibodies, no manufacturers currently have the expertise to produce mucins. One issue to address is that the physical properties that make mucins so useful—being gel-like as well as a specific type of sticky—make them especially challenging to produce at scale because they gum up the downstream bioprocessing.

Even setting production difficulties aside, some researchers have doubts that mucins will completely soothe xerostomia. On their own, mucins “are definitely not enough,” Pedersen of the University of Copenhagen says. “But we need to start somewhere, and mucins are definitely what we need to work on most.” Her team is studying the lipids in saliva, and she believes adding those into the mix would make an artificial saliva product more effective.

## Supercharging saliva

People with xerostomia may benefit from a substance that is simpler, cheaper, and easier to commercialize than mucins and mucin analogs, some researchers say—especially if researchers could develop a material that improves people’s remaining saliva. According to Prashant Sharma, a tribologist at the University of Groningen, one property to play with is electrical charge. Mucins, like most molecules in the natural world, are negatively charged, he reasoned, so a positively charged coating in the mouth could attract what saliva is already in there.

His team created a material that combined two naturally occurring compounds widely used in the pharmaceutical industry—chitosan, a slightly cationic polysaccharide, and catechol, an adhesive molecule. In lab studies, the substance draws mucins toward it and holds them in place. “It is like a primer layer, attaching itself and recruiting useful molecules from saliva,” Sharma says. Although he has patented the material, it has not yet been tested in animals.

At the University of Leeds, food chemist Sarkar is also working on a relatively simple substance that can alleviate xerostomia. She and her colleagues created positively charged microgels—microscale hydrogels—containing a protein called lactoferrin. They then dispersed the microgels within larger-scale hydrogels containing carrageenan, a negatively charged, seaweed-derived polysaccharide. The opposite charges in the combined material pull the two components together to create a lubricity similar to that of saliva, and the microgels essentially act as a sponge, giving the material its long-lasting water-releasing and lubricious properties. “We are trying to replicate the structure of saliva” without the hassle of recreating its components, Sarkar says. The team reported the material in 2020 and is now comparing its properties with those of artificial saliva products and exploring the feasibility of manufacturing it at a larger scale.

Ideally, she says, topical therapies made from sustainable and safe food-grade materials would help most people who need such products. “For us, the main question is, Do we really want to recreate saliva? Or [can we] replicate the properties that are useful so you can eat properly and have an excellent quality of life?”

## Alla Katsnelson is a freelance contributor to

Chemical & Engineering News, *the independent news outlet of the American Chemical Society.*

